# Pet dogs *(Canis lupus familiaris)* release their trapped and distressed owners: Individual variation and evidence of emotional contagion

**DOI:** 10.1371/journal.pone.0231742

**Published:** 2020-04-16

**Authors:** Joshua Van Bourg, Jordan Elizabeth Patterson, Clive D. L. Wynne

**Affiliations:** Department of Psychology, Arizona State University, Tempe, Arizona, United States of America; Middlesex University, UNITED KINGDOM

## Abstract

Domestic dogs have assisted humans for millennia. However, the extent to which these helpful behaviors are prosocially motivated remains unclear. To assess the propensity of pet dogs to actively rescue distressed humans without explicit training, this study tested whether sixty pet dogs would release their seemingly trapped owners from a large box. To examine the causal mechanisms that shaped this behavior, the readiness of each dog to open the box was tested in three conditions: 1) the owner sat in the box and called for help (distress test), 2) an experimenter placed high-value food rewards in the box (food test), and 3) the owner sat in the box and calmly read aloud (reading test). Dogs were as likely to release their distressed owner as to retrieve treats from inside the box, indicating that rescuing an owner may be a highly rewarding action for dogs. After accounting for opening ability, dogs released the owner more often when the owner called for help than when the owner read aloud calmly. In addition, opening latencies decreased with test number in the distress test but not the reading test. Thus, rescuing the owner could not be attributed solely to social facilitation, stimulus enhancement, or social contact-seeking behavior. Dogs displayed more stress behaviors in the distress test than in the reading test, and stress scores decreased with test number in the reading test but not in the distress test. This evidence of emotional contagion supports the hypothesis that rescuing the distressed owner was an empathetically-motivated prosocial behavior. Success in the food task and previous (in-home) experience opening objects were both strong predictors of releasing the owner. Thus, prosocial behavior tests for dogs should control for physical ability and previous experience.

## Introduction

Prosocial behaviors occur when individuals voluntarily act to benefit one or more individuals other than themselves [1]. Although most authors describe prosocial behaviors in terms of costs and benefits (e.g., [2]), some definitions require that the prosocial actor demonstrate concern for the receiver and thus, an understanding of the receiver’s emotional state [3, 4]. However, the extent to which empathy and sympathy shape prosociality in non-human animals remains unclear [5, 6].

Prosocial behaviors are generally regarded as widespread among species [7, 8]. Although prosociality has been experimentally demonstrated in corvids, parrots, canids, rodents, and social insects (for review, see [9]), research on prosociality in non-human animals has primarily focused on primates [10, 11]. However, some of the most commonly proposed evolutionary drivers are relatively uncommon in primates compared to other taxa [9]. Thus, a broader phylogenetic framework is needed to clarify the evolutionary origins of prosocial behavior [12, 13].

Canids may serve as a valuable taxon for such investigations given that a number of canid species are highly social and may cooperate in territory defense, group hunting, and allomaternal care [14, 15]. Studies on domestic dogs in particular, may afford a unique perspective on the mechanisms shaping the evolution of prosocial behavior as domestication and selective breeding may have favored hypersocial tendencies in dogs [16]. Dogs have also been presented as a model for the evolution of human social cognition [17]. Furthermore, pet, shelter, and free-ranging dogs differ greatly in their experiences with humans and overall social ecology. Therefore, intraspecific comparisons of dogs may yield insight into the relative importance of environment and rearing on the expression of prosocial behaviors [18]. Finally, as almost all dogs share some component of their ecological niche with humans and millions form intimate associations with their owners [19], studies focusing on dogs also provide a rare opportunity to examine prosociality in a naturalistic, interspecific context [20].

Previous work has established that dogs are sensitive to human attentional states [21, 22] respond discriminately to human visual and auditory emotional signals (see [13] for review) and provide emotional comfort to humans [23]. For example, Custance and Mayer [24] found that dogs were less likely to approach a person humming a song than a person feigning a distressed state by pretending to cry. In addition, physiological and behavioral data suggest that dogs and owners synchronize their emotions [25, 26]. Thus, dogs are an ideal focal species for research on empathetically-motivated prosocial rescue.

Prior studies on prosocial behavior in dogs have yielded mixed results. Macpherson and Roberts [27] found that dogs did not seek help from a human bystander when the dog’s owner pretended to have a sudden heart attack or when a bookcase appeared to fall on top of the owner. However, dogs were more attentive towards the owner in these emergency scenarios than in a control condition, which may indicate concern in the dogs. Kaminski et al. [28] tested whether dogs used gaze alternation to direct a human towards a hidden object with which the human had previously interacted. Their findings suggested that dogs concentrated on objects that they wanted for themselves rather than the object desired by the human.

Bräuer et al. [29] found that dogs pushed a button to open a door that separated their owners from an object when the owner pointed directly to the button or signaled their desire for the object in a naturalistic manner. However, it is unlikely that this behavior constituted prosocial helping because the owner directed the dog’s attention towards the mechanism rather the goal [9]. Additionally, the owner’s vocalizations and actions were not controlled in the “Natural” treatment and thus stimulus enhancement and increased arousal may explain the increased frequency of button pushing relative to the control conditions [20; 30].

Quervel-Chaumette et al. [20, 31] found that dogs preferentially donate food to familiar dogs but not to humans, regardless of the human’s identity. However, as the humans were prohibited from communicating with the dogs, this situation may have been aversive to the dogs, which might have impeded or precluded prosocial food donation.

Prosocial helping paradigms have typically employed an ‘out-of-reach task’ in which potential donor individuals can choose to retrieve and transfer an item to a recipient individual, for whom this item is otherwise out of reach (e.g., [32]). The current study adopted an alternative test for helping that has been used to successfully demonstrate prosocial rescue behavior in rats [33] and ants [8]. In this paradigm, a potential rescuer is free to move about a testing arena while a conspecific in the same arena is either trapped inside a restrainer apparatus that can only be opened from the outside (rats), or partially buried in sand and tied down by a nylon thread (ants).

Rescue behavior in the trapped-other test may be attributed to the rescuer’s selfish desire for social-contact with the trapped individual rather than emotional contagion [9]. As in previous tests of prosociality, rescuing in this paradigm may also result from social facilitation and stimulus enhancement [34]. Moreover, rescuing may be a by-product of the rescuer’s close proximity to the restraining mechanism, regardless of the rescuer’s motivation for approaching the trapped individual [35]. However, studies manipulating the affectual state of the trapped individual may be able to detect empathetically-motivated prosocial behavior by controlling for these alternative explanations for apparent prosocial behavior.

To date, two studies have tested for prosocial rescue behavior in dogs. Sanford et al. [36] used a modified version of the trapped-other paradigm to assess whether dogs would walk to their owner in an adjacent room, which required pushing through a small, windowed door. In the experimental condition, the owner pretended to cry and in the control condition, the owner hummed. More recently, Carballo et al. [37] tested whether dogs would release their seemingly distressed owners from a wooden box. In each of two experiments, owners assigned to the experimental condition were asked to convey distress in a naturalistic manner. In the control condition of the first experiment, owners used their phone or read a book. In the control condition of the second experiment, owners were also instructed to periodically call to their dogs from inside the box. Each dog was assigned to a single condition and was tested in three consecutive trials.

These studies yielded limited evidence that opening the door constituted a prosocial behavior. Sanford et al. [36] observed interesting trends in post-hoc comparisons but the primary findings of this study did not support the hypothesis that dogs prosocially rescued their owners. Considering only individuals that opened, dogs in the experimental condition opened faster. However, in the analysis of all dogs, opening latencies did not differ between groups. More importantly, opening frequencies in the experimental (7/17) and control (9/17) groups did not differ.

Carballo et al. [37] found that dogs in the experimental group released their owners more readily and faster than did dogs in the control group, but these findings were limited by uncontrolled variables. Most notably, in the unscripted experimental condition owners were allowed to use gestures and vocal commands other than calling to the dog to elicit opening. Thus, social facilitation and obedience may explain the effects of test condition observed in this study.

Evidence that the owner’s distress increased the stress of the dog in Carballo’s [37] and Sanford’s experiments [36] was inconsistent. Therefore, it remains unclear whether emotional contagion leads to rescue behavior in dogs. Carballo et al. found significant effects of test condition on only one of five stress measurements while Sanford observed interesting trends but did not find significant effects of test condition on stress. Specifically, Sanford hypothesized that successful dogs in the experimental group suppressed a distress response in order to rescue the owner. However, they did not find evidence of this hypothesized distress response.

Tests for prosocial behaviors in dogs must control for ability and previous experience in order to accurately assess prosocial tendencies. Warneken & Tomasello [38] posited that instrumental helping behaviors incorporate a motivational component as well as a cognitive component in which the donor both recognizes the recipient’s goal and understands how to fulfill that goal. Previous studies have demonstrated that dogs may have difficulties understanding the intentions and goals of humans in instrumental helping paradigms [28, 29]. Thus, dogs that fail to complete a prosocial task may lack the requisite ability, rather than prosocial motivation.

Given that Sanford [36] and Carballo [37] did not control for task ability, it remains unclear whether dogs prosocially rescue their owners. Moreover, these studies deployed between-subjects designs and neither study assessed whether the dog understood how to open the door. Thus, the findings of Carballo and Sanford may reflect differences between dogs assigned to different conditions in the ability to open the door to their owner rather than the effect of the owner’s distress on the motivation of the dog.

In summary, prior tests for prosocial behaviors in dogs have been hindered by a pervasive challenge—demonstrating that dogs perceive the contingencies of the prosocial task [9]. For thousands of years, dogs have been trained to assist humans with tasks such as herding and guarding livestock [39], yet the extent to which dogs understand the consequences of these helpful behaviors, as well as their underlying motivations for performing these actions, remain unclear [29].

To address these limitations, we presented 60 dogs with a series of three tests. To assess the propensity of pet dogs to rescue their owners, we tested whether dogs would move a piece of foam-board restraining their seemingly distressed owner inside of a box (the “restrainer apparatus”). To examine the relative contributions of stimulus enhancement, desire for social contact, and empathetic concern in motivating rescue behavior in this paradigm, we tested whether each dog would display the same propensity for opening the door when the owner calmly read aloud from within the restrainer apparatus. In a third test, we placed high-value food rewards in the apparatus to determine whether the dog was capable of moving the restrainer door when sufficiently motivated.

To further examine the role of previous experience and ability in determining whether the dog opened the apparatus, we conducted a short survey after the dog completed the tests. To examine the possibility of emotional contagion, we also compared across test conditions the number of stress behaviors exhibited by the dog. In order to account for the staged nature of the rescue scenario, we rated the sincerity of the owner’s distress vocalization and analyzed whether the owner’s convincingness affected the dog’s performance. Finally, to assess whether dogs attempted to solicit assistance for their owner, we recorded the latency of the dog to approach the doorway through which the experimenter exited the room, as well as the frequency with which the dog returned to this location during each test.

## Materials and methods

This research was approved by the Arizona State University Institutional Animal Care and Use Committee (*Protocol Number*: *18-1620R*).

### Subjects

Sixty-seven pet dogs (*Canis lupus* familiaris) of diverse and mixed breeds were volunteered by their owners to participate (S1 Dataset). Only dogs over 9 months of age were recruited for this study. There were no other predetermined criteria for study subjects. Seven dogs were excluded from analyses: two dogs would not leave the room with the experimenter, one owner did not follow the script, one dog’s leash unraveled and accidentally opened the door, one dog opened the apparatus before the owner started vocalizing, and in one case the video cameras malfunctioned. To prevent satiation, all dogs were fasted in the four hours proceeding the experiment.

### Layout

**Fig 1:**
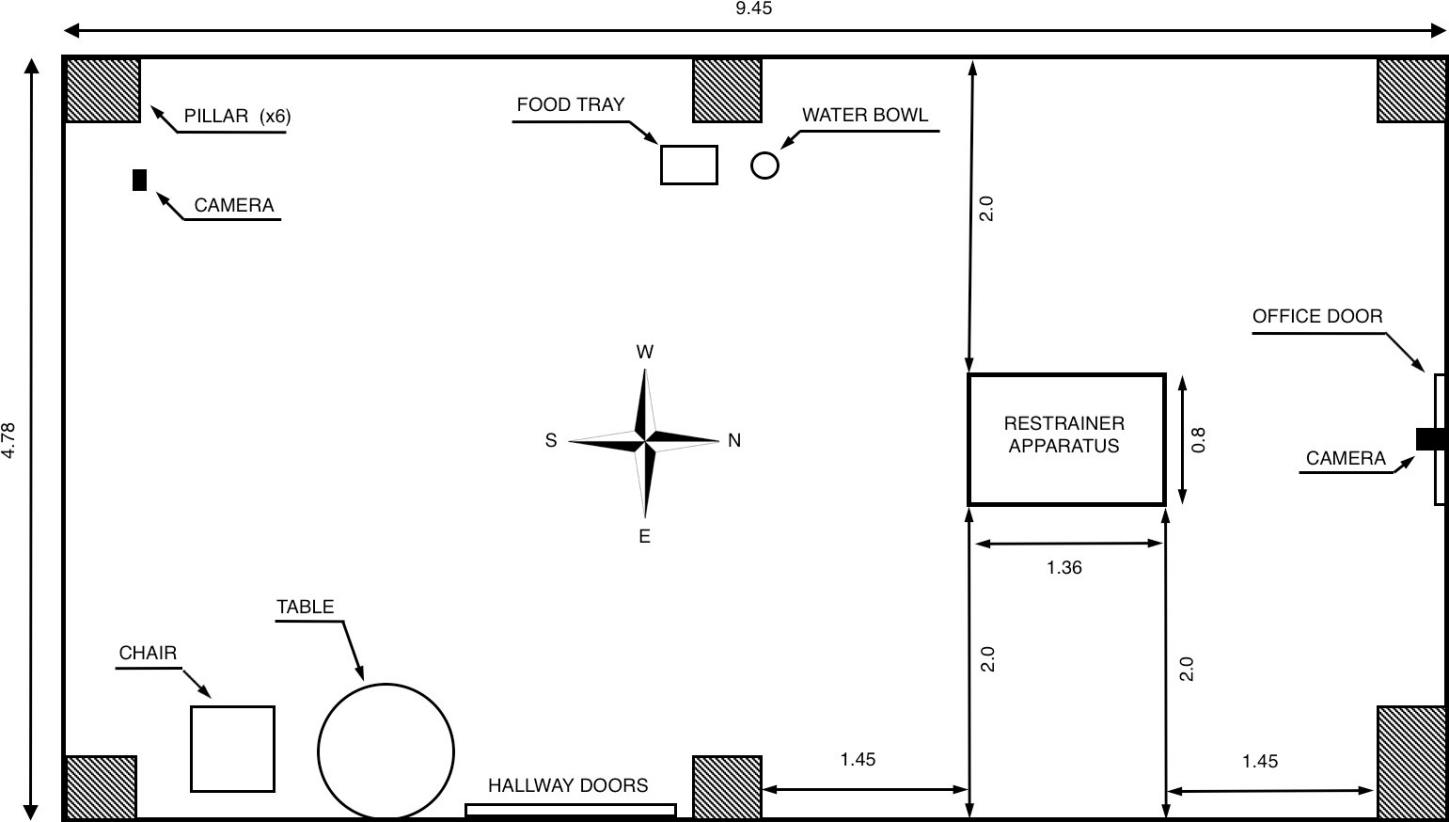
Schematic of the experimental setup. Layout of the room in which the experiment was carried out. All dimensions are in meters.

The restrainer apparatus was centered in the northern half of a 9.45m x 4.78m testing room, in line with a camera mounted above a door leading to an adjacent office (Fig 1). A table and a chair facing the southeast corner of the room sat next to a set of doors on the east wall, which led to an adjacent hallway. A water bowl and tray were centered along the west wall and a tripod-mounted camera stood in the southwest corner of the room.

### Apparatus

**Fig 2:**
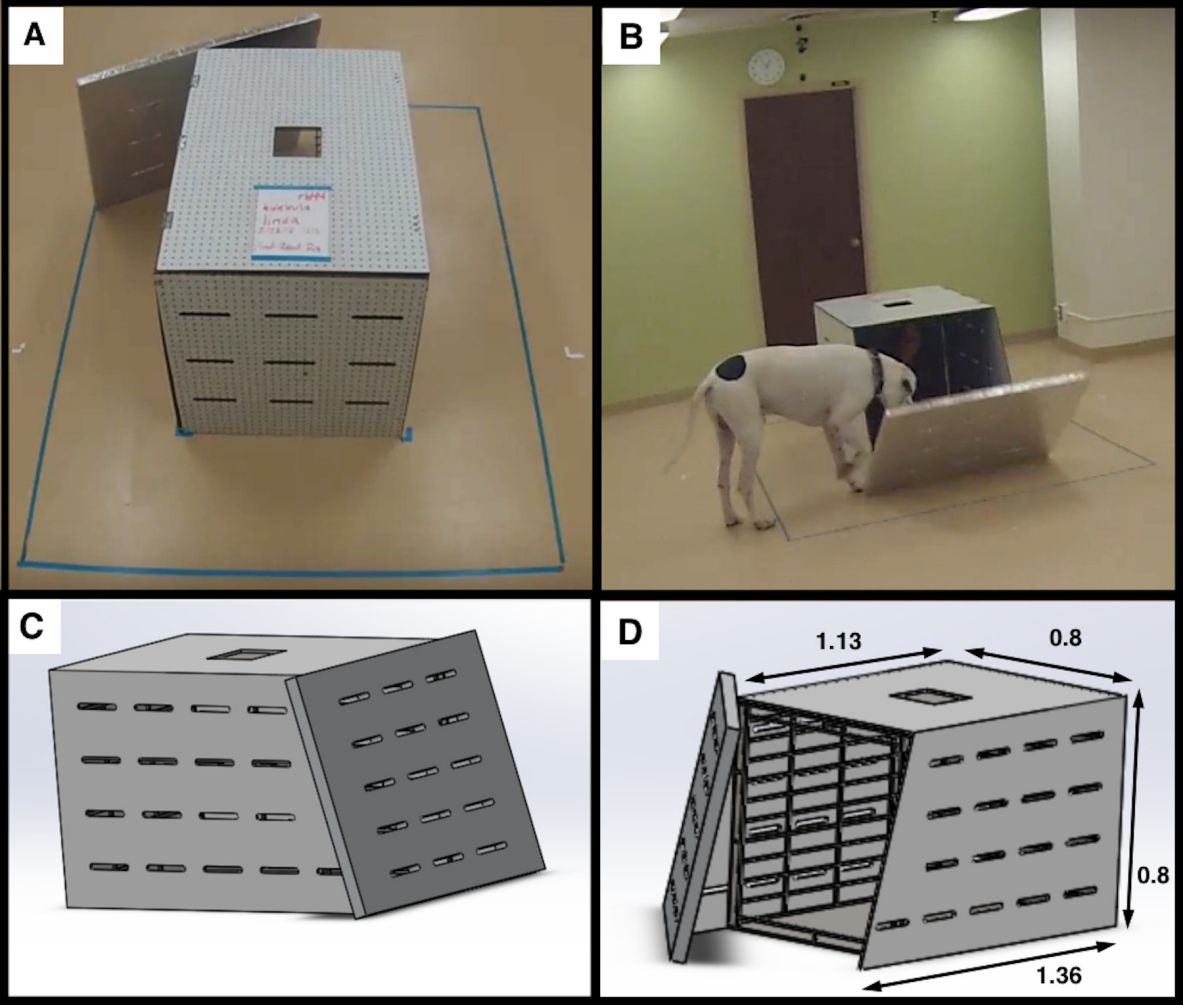
Restrainer apparatus. A. View from north camera. B. View from south camera. C. Rendering of the apparatus when closed. D. Schematic of the apparatus when open (dimensions are in meters).

The restrainer apparatus was constructed using acrylic-coated peg board supported on a 0.8 x 0.8 x 1.0m metal dog kennel with the front face removed (Fig 2). The sides of the apparatus were angled from 1.36m in width at the bottom to 1.13m at the top to allow the restrainer door, an aluminum tape-coated foam board, to rest against and seal the front opening of the apparatus. The door was wider than the apparatus and weighed only 670g, so that it could be easily moved by dogs of all sizes. A 12cm^2^ hole was cut into the top of the restrainer apparatus to allow food to be dropped inside without opening the restrainer door. To increase visibility into the apparatus while still preventing physical contact, five rows of horizontal slits (2 x 20cm) were cut into each side of the apparatus and the door spaced 10-cm apart vertically and horizontally.

### Procedure

In each step of the pre-test acclimation phase, the restrainer door remained open. Upon entering the testing space, each dog was allowed to freely explore the room while the experimenter (E) reviewed the protocol with the owner. To ensure that the dog was comfortable with handling and with leaving the room, E then led the dog out of the testing room and down the adjacent hallway. Upon returning, E instructed the owner to guide the dog around the perimeter of the testing room and restrainer apparatus. With the dog and owner standing in front of the apparatus, E then moved the restrainer door perpendicular to the long axis of the apparatus for four seconds by lightly tapping or pushing. This exposed all dogs to the sound of the aluminum tape scraping the floor before the first test. E then directed the owner to a chair facing the wall in the far corner and then exited the room. Each dog was given at least three additional minutes to fully acclimate to the testing room and restrainer apparatus while the owner sat silently reading, ignoring the dog. After the acclimation period was completed, E lead the dog out of the testing room and down the hallway. This allowed an assistant to enter from an adjacent office, unseen by the dog, to prepare the testing room and owner for the upcoming test.

The dog was then given a series of three tests–the primary test for prosocial rescue behavior (distress: D), a social facilitation control test (reading: R), and an assessment for task ability (food: F). Ten dogs were randomly assigned to each of the six possible orders of the three tests. To rule out the possibility that random assignment did not adequately control for physical differences among test subjects, separate linear models were used to test the regression of test order (DFR, DRF, FDR, FRD, RDF and RFD) on the age, height and weight of the dog. Likewise, a general linear model was used to test the regression of test order on the sex of the dog.

Once the testing room and owner were prepared, E returned with the dog and announced the beginning of the distress test. E then released the dog and exited into the hallway, leaving the dog alone with the owner in the testing room. For the duration of the test, E observed through an observation window in the hallway door.

Once the hallway door was fully closed, the owner tapped the bottom of the restrainer apparatus four times before vocalizing distress using only the words ‘help’ or ‘help me.’ Tapping the floor ensured that the dog’s attention was directed to the apparatus immediately in all test conditions. In advance of the test, the owner was instructed to convey distress as genuinely as possible while maintaining a consistent volume and even pace. The dog was given up to two minutes to release the owner from the apparatus by dislodging the restrainer door. If the dog rescued the owner, the owner ceased distress vocalizations and immediately began to pet and praise the dog for up to 30 seconds. If the dog did not rescue the owner, E announced the end of the test and the owner ceased vocalizations. After the test, E led the dog out of the room and down the hallway while the owner remained in the restrainer apparatus.

In the reading test, the owner read aloud from a magazine while conveying a calm and relaxed state. In advance of the test, the owner was instructed to match the volume and pace of his narration to his distress vocalizations so that only his tone differed between the two tests. To facilitate this consistency, a sound level monitor was placed inside the apparatus and adherence to the instructions was later confirmed from recordings of the tests. In all other regards, the reading and distress tests were conducted identically.

To assess the ability of the dog to move the restrainer door and access the inside of the apparatus, food was placed inside the apparatus for the third task. The owner did not participate in the food test but remained in the testing room to prevent the dog from becoming distressed by the owner’s absence. To minimize distractions, the owner sat in the same chair used during habituation and again faced the wall while ignoring the dog.

Before E returned with the dog to begin the test, the assistant set out a plate with food rewards on a table near the hallway entrance. These rewards consisted of four pieces of the dog’s normal dry kibble with a 1cm^3^ piece of hot dog. In addition, the assistant placed two identical trays on the floor—one outside of the apparatus (demonstration tray) and the other inside the apparatus.

Once the assistant was quietly situated in the adjacent office, E returned with the dog. Upon re-entering the testing room, E collected a food reward from the table, led the dog to the demonstration tray, and dropped the food reward into the tray. After allowing the dog to retrieve the food, E repeated this demonstration with a second food reward. These demonstrations provided confirmation that each dog was sufficiently motivated by food and permitted the formation of an association between the actions of E, the sound of treats hitting the tray, and the consequent reward.

In the test, E dropped a food reward inside the apparatus, released the dog, and exited into the hallway. The dog was then allowed up to two minutes to open the apparatus before E ended the test. If the dog successfully opened the apparatus and retrieved the treats, the dog was permitted an additional 30s for exploratory behaviors. E then re-entered the testing space and announced the end of the test before leading the dog out into the hallway.

Following the conclusion of the third test, the owner was given a short survey on the previous experience and behavior of the dog relevant to the experiment.

### Measurements

#### Opening the apparatus

Opening the apparatus in all three tests was defined by the same criteria—contact between the apparatus and any part of the dog other than the tail which resulted in the restrainer door moving away from the apparatus. In addition to the binary outcome of whether the dog opened the apparatus, the latency (in seconds) to open the apparatus was also recorded. A latency of 120 seconds (the maximum length of the test) was assigned to tests in which the dog did not open the apparatus. Latencies were natural log-transformed to reduce skew and kurtosis.

In three reading, two distress and one food test, the dog nudged the restrainer door but did not enter the apparatus. These tests were allowed to continue until the dog entered the apparatus or two minutes elapsed. However, in all analyses the same criteria for opening and latency were applied to these tests.

#### Stress behaviors

To assess the level of stress and activity of the dog, a composite stress score was generated using four positive behavioral indicators (running, barking, whining, and sniffing the apparatus) and one negative indicator (laying down). The occurrence of each behavior was treated as a binary outcome and all behaviors were weighted equally such that the composite score increased by an absolute value of one for each additional behavior exhibited by the dog. Thus, composite scores ranged from negative one for an inactive and unstressed dog to positive four for a very stressed and active dog.

#### Approaching the hallway

To assess whether dogs that failed to open the apparatus sought out the experimenter for assistance, the latency and frequency with which the dog approached a rectangular floor area extending 0.5 meters from the hallway doors were recorded. Hallway approaches were tallied each time the dog contacted this area with any part of the body other than the tail (see S1 Text).

#### Video coding

All measurements were recorded from video recordings using the event-logging software, BORIS [40] by six coders who were blind to the nature of the study. Coders were trained to 100% agreement using six randomly selected tests. For each measurement, one coder scored all tests and at least half of the data were scored by a second coder. For all tests, the coders agreed on whether the dog opened the apparatus and all opening latencies were consistent to within 0.1 seconds. For stress scores, hallway approaches and hallway latencies, any discrepancies were reconciled by a third coder.

#### Owner distress vocalizations

To assess whether the convincingness of the owner’s distress affected the performance of the dog, audio recordings of the owner’s distress vocalizations were rated on a scale of perceived sincerity from one to six. To provide a consistent metric of sincerity, all distress vocalizations were rated by one coder. Up to three intervals per test were rated to produce an average vocalization score. When the duration of the test was at least 30 seconds, the first, middle, and last ten seconds of the test were rated. When the duration of the test was between 20 and 30 seconds, the first and last 10 seconds of the test were rated. When the duration of the test was less than 20 seconds, playback of the entire test was given a single rating.

#### Survey on previous experience

The survey of the dog’s previous exposure to relevant scenarios consisted of four yes-or-no questions: (1) Has your dog ever seen you in genuine emotional or physical distress? (2) Has your dog ever heard you say "help" or “help me”? (3) Has your dog ever played hide-and-seek? (4) Has your dog ever opened boxes, bins, cabinets, doggy gates, garbage cans or other objects?

### Analysis

Data were analyzed in R version 3.4.1. Models were tested using the package “lme4” [41] and post-hoc comparisons were conducted using the package “multcomp” [42]. To analyze repeated measures, mixed effects models were constructed with study subject treated as a random intercept and all remaining predictors treated as fixed effects. Fixed effects models were used to analyze non-repeated measures.

To analyze continuous outcomes and discrete numerical outcomes with a sufficient number of levels, linear regression analyses were conducted using linear models (LMs) and linear mixed models (LMMs). P-values were then obtained using Wald tests (α = .05 for each coefficient). To analyze binomial outcomes, logistic regression analyses were conducted using generalized linear models (GLMs) and generalized linear mixed models (GLMMs). P-values were then obtained from likelihood ratio tests (LRTs) of nested models. Specifically, the difference between the full model and the null model without the effect of interest was tested using the Chi square distribution (α = .05 for each predictor).

To test the assumptions of each model, residual plots were inspected to confirm linearity, Levene’s tests were conducted to confirm homoscedasticity, and standard normal quantile plots were inspected to confirm that residuals were normally distributed. Model assumptions were met in all analyses described herein.

## Results

### Frequency of opening

Of the 60 dogs tested, 20 rescued the owner in the primary distress test, 19 successfully retrieved treats from the apparatus in the food control task, and 16 released the owner from the apparatus in the reading test. To test whether releasing the owner from the apparatus in the distress test could be attributed to social-contact seeking behavior, the frequencies of opening in the distress and reading tests were compared using a logistic regression analysis. A general linear mixed model (GLMM) was constructed with opening modeled as a binomial outcome, study subjects treated as a random intercept, and test condition treated as a fixed-effect with two levels (distress and reading). To test for effects of repeated testing, test number was included in the model as a fixed effect with three levels (first, second, and third test). To account for the ability of the dog to open the apparatus, the outcome of the food control task was also incorporated into the model of rescuing the owner as a fixed-effect predictor with two levels (failure and success).

**Fig 3:**
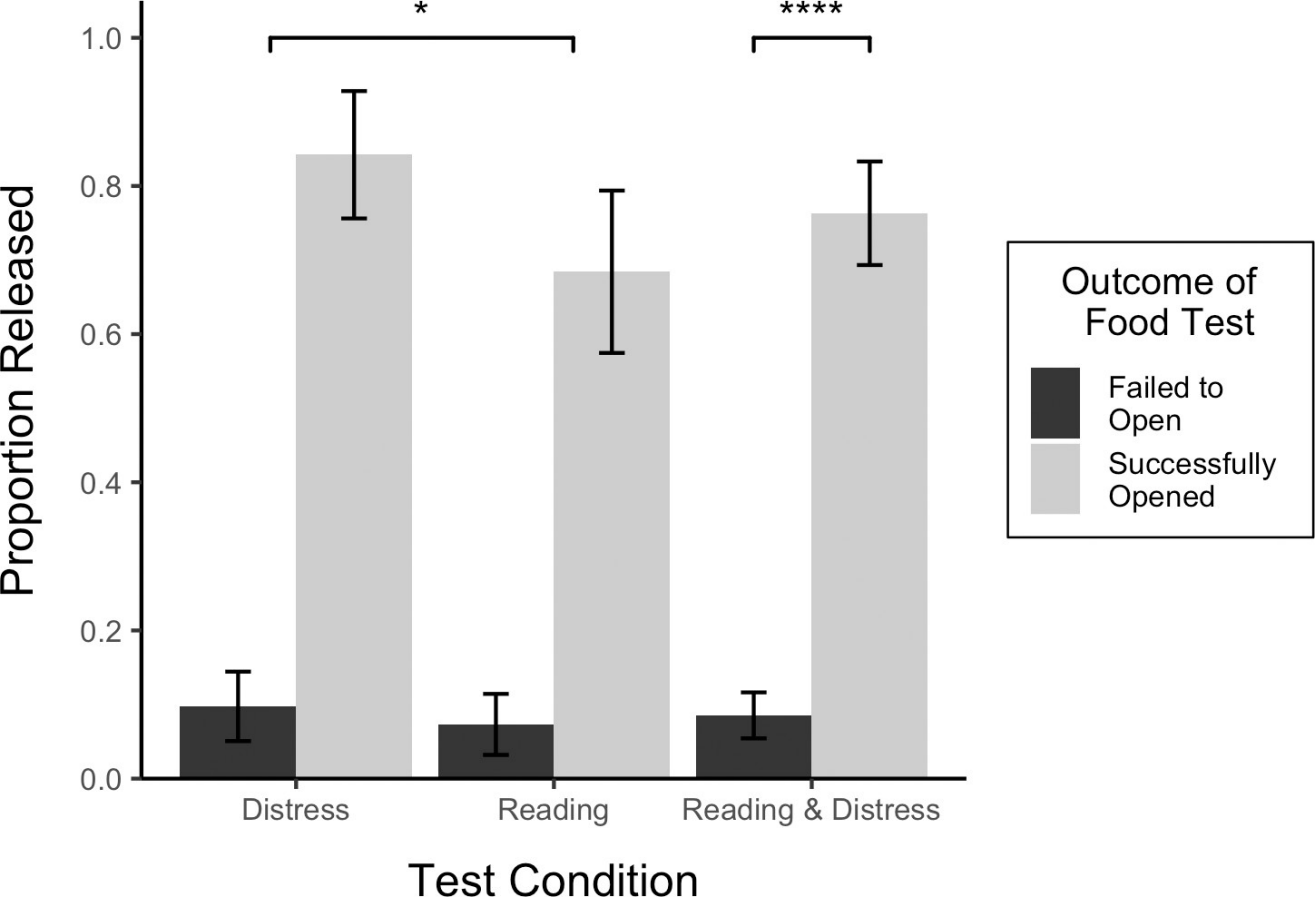
Proportion of owners released in the distress and reading tests by outcome of the food test. The distress test is shown in left cluster of bars, the reading test, in the middle cluster, and the distress and reading tests are combined in the right cluster. Error bars show standard errors.

Dogs that successfully retrieved food from the apparatus during the food test were significantly more likely to release the owner (29 of 38 distress and reading tests) than were dogs that failed to retrieve the food (7 of 82 distress and reading tests), 𝛸 ^2^(1, *N* = 60) = 36.35, *p* < .0001 (Fig 2). The main effect of test number was not significant, 𝛸^2^(2, *N* = 60) = 1.80, *p* > .05. Opening rates in the distress and reading tests were similar, both among dogs that successfully retrieved food (distress = 16/19, reading = 13/19) and dogs that failed to retrieve food (distress = 4/41, reading = 3/41). However, after accounting for test number and performance in the food test, the effect of test condition was significant, 𝛸^2^(1, *N* = 60) = 8.67; *p* < .01 (Fig 3). The random effect of test subject was significant 𝛸^2^(1, *N* = 60) = 31.46, *p* < .0001.

### Latency to open

To assess whether dogs freed the owner faster in the distress test than in the reading test, a linear regression analysis of latency to open the apparatus was conducted using a linear mixed model (LMM). A latency of 120 seconds (the length of the test) was assigned to tests in which the dog did not open the apparatus. Latency data were natural log-transformed to reduce skew and kurtosis. To control for task ability, only dogs that demonstrated the capacity to open the apparatus in the food control task were included in this analysis. To assess whether potential learning and desensitization effects differed among conditions, test number and the interaction between test number and condition were included in the model as fixed effect predictors. Study subject was treated as a random effect.

**Fig 4:**
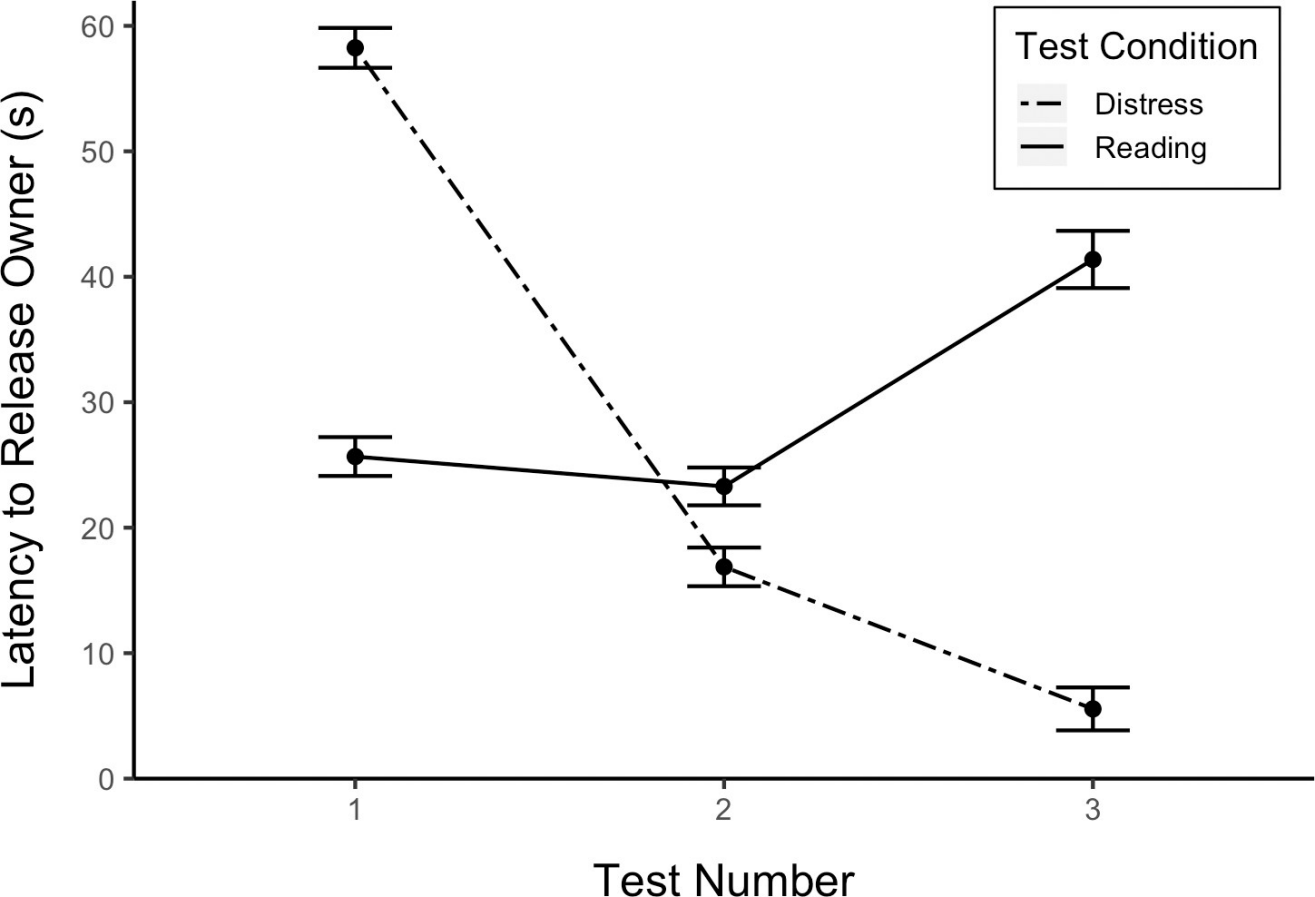
Latency to release the owner by test condition and number (among dogs that successfully opened in the food test). Latency means and standard errors displayed here are back-transformed from the natural log transformation used in analysis. Error bars show standard errors.

The main effect of test number was significant, 𝛸^2^(2, *N* = 19) = 9.62, *p* < .01. The main effect of test condition was not significant but dogs tended to open the apparatus more quickly in the distress test than in the reading test, 𝛸^2^(1, *N* = 19) = 3.60, *p* < .10. However, the interaction between test condition and number was significant, 𝛸^2^(2, *N* = 19) = 8.83, *p* < .05 (Fig 4). Specifically, latency to release the owner decreased with test number in the distress test but not in the reading test.

### Survey on previous experience

**Fig 5:**
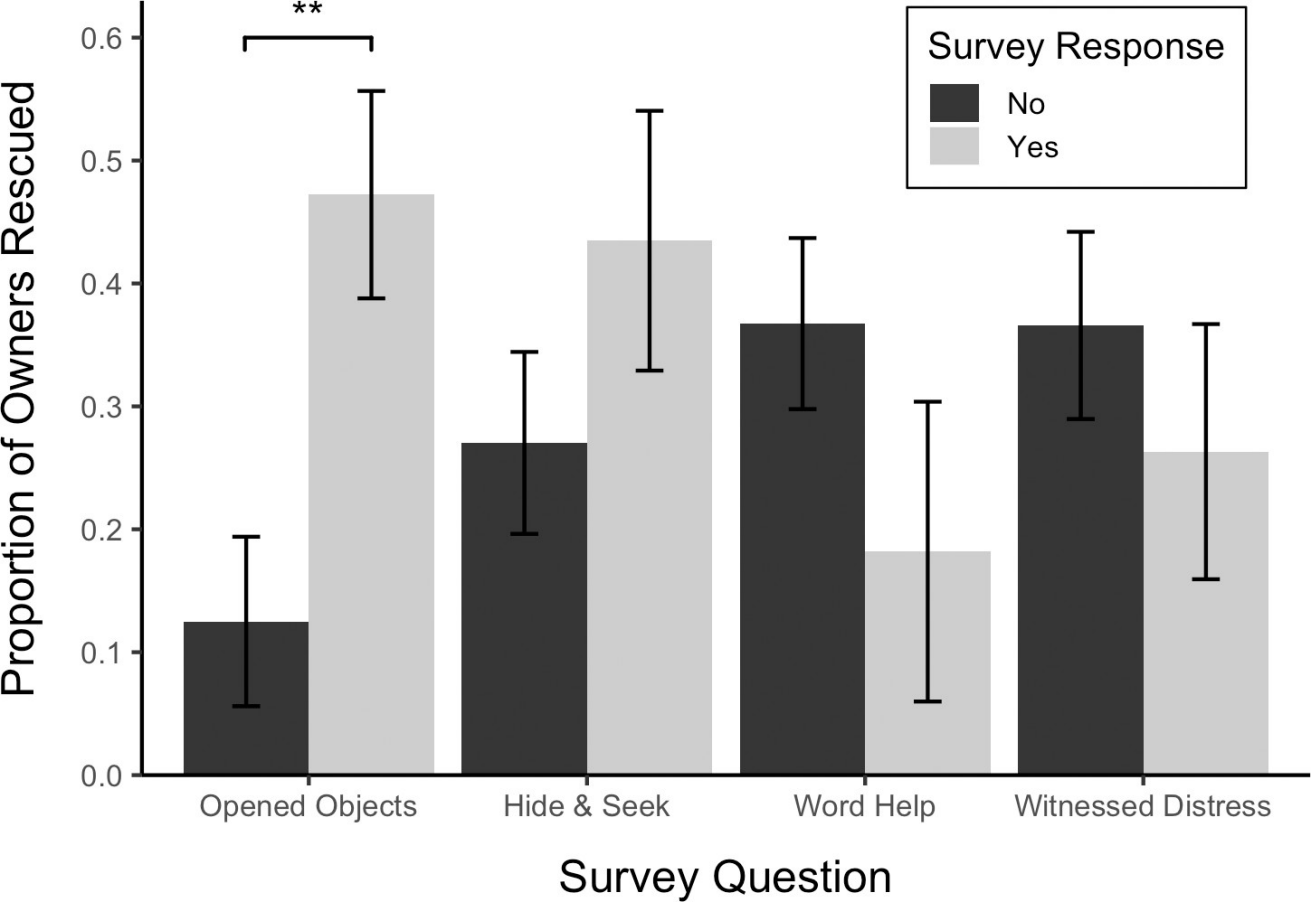
Proportion of owners rescued by survey response. Error bars show standard errors.

To assess the role of previous experience in predicting whether the dog rescued the owner, a logistic regression analysis of opening in the distress test was conducted using a GLM in which each of the four survey responses was treated as a binary, fixed-effect predictor. Whether the dog previously played hide-and-seek, 𝛸^2^(1, *N* = 60) = 0.84, *p* > .05, or heard the word “help”, 𝛸^2^(1, *N* = 60) = 1.52, *p* > .05, did not predict whether the dog rescued the owner. Dogs that had previously seen the owner in genuine distress tended to open the apparatus less frequently but this effect was not significant, 𝛸^2^(1, *N* = 60) = 2.83, *p* < .1. However, when the owner reported that the dog had experience opening objects, the dog was four times more likely to open the apparatus, 𝛸^2^(1, *N* = 60) = 7.50, *p* < .01 (Fig 5).

### Stress and activity

To test the hypothesis that the distress expressed by the owner increased the level of activity and stress displayed by the dog, a linear regression analysis of composite stress scores was conducted using a LMM. To test for acclimation and sensitization across repeated tests, and to assess whether these effects differed among conditions, coefficients for test number and the interaction between test number and condition were included in the model. Subject intercepts were included in the model as a random effect. Linear hypotheses were then specified to compare the distress test to the reading test and the distress test to the food test. P-values for these pairwise comparisons were adjusted (such that α = .05) using the “single-step” method which accounted for the joint normal distribution of each linear function.

**Fig 6:**
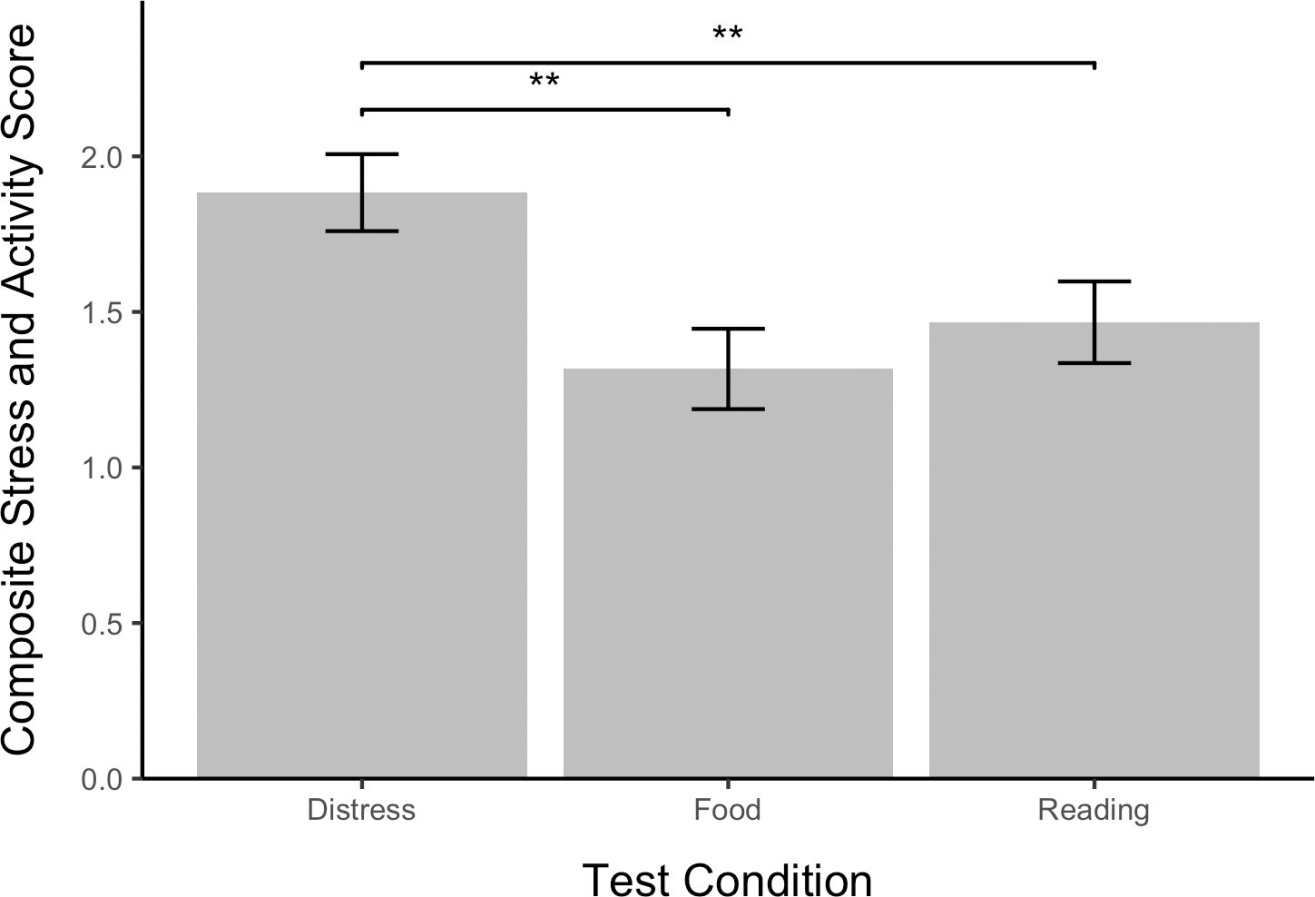
Stress and activity level by test condition. Positive and negative behavioral indicators of stress and activity were used to generate a composite score for each dog in each test. The occurrence of each behavior was treated as a binary outcome and all behaviors were weighted equally. Error bars show standard errors.

**Fig 7:**
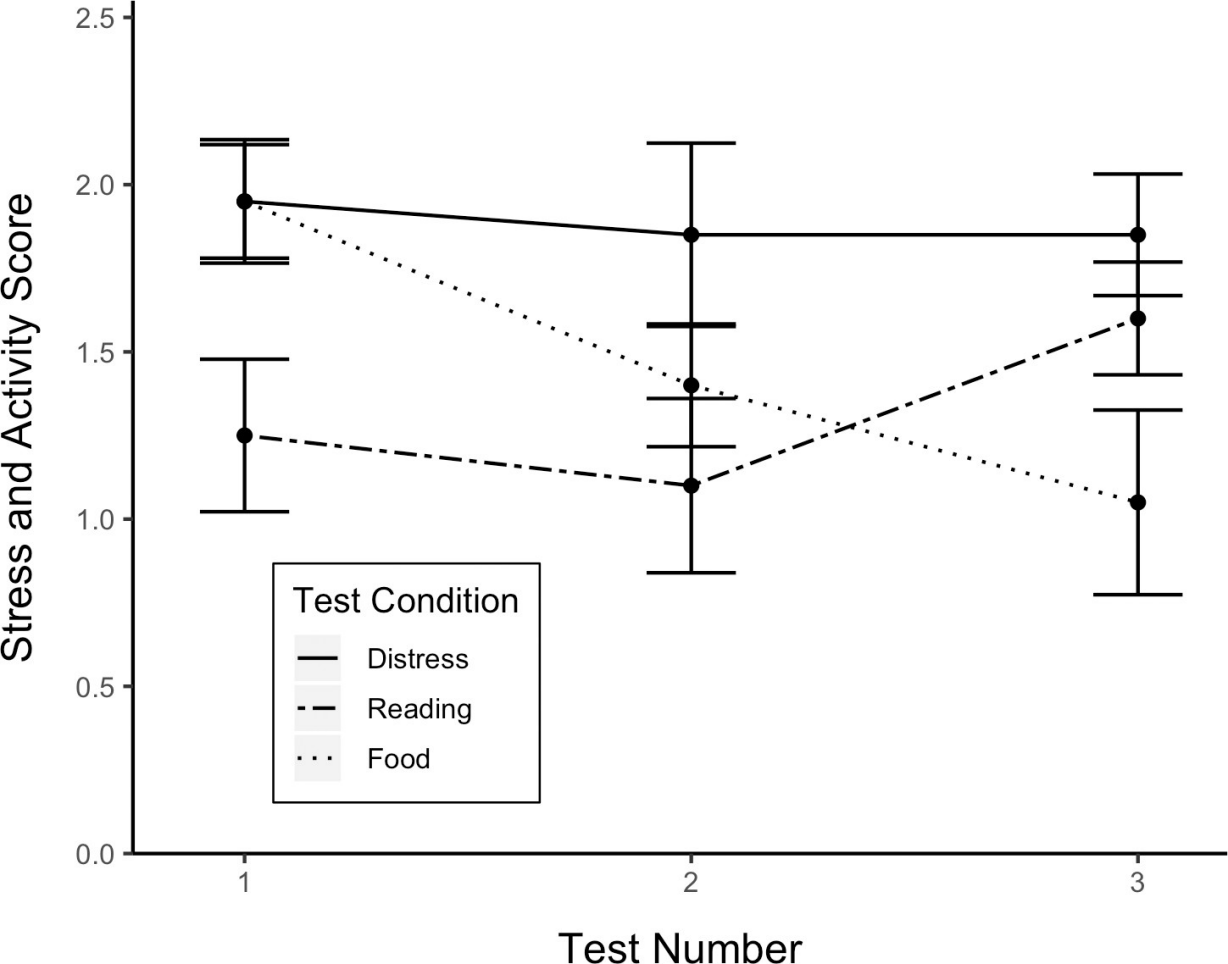
Stress and activity score by test number and condition.

The main effect of test condition was significant, 𝛸^2^(2, *N* = 60) = 15.74, *p* < .001. Dogs were more active and displayed more stress behaviors in the distress test ($\bar{x}$ = 1.89, *SE* = .12) than in either the reading test ($\bar{x}$ = 1.47, *SE* = .13) *z* = 2.82, p < .01, or the food task ($\bar{x}$ = 1.32, *SE* = .13) *z* = 3.83, *p* < .01 (Fig 6). The main effect of test number was not significant, 𝛸^2^ (2, *N* = 60) = 0.87, *p* > .05. However, there was a significant interaction between test number and condition, 𝛸^2^ (2, *N* = 60) = 12.68, *p* < .01, whereby stress scores decreased with test number in the reading test but not in the distress or food test (Fig 7). The effect of study subject was significant, 𝛸^2^ (1, *N* = 60) = 15.52, *p* < .0001.

### Approaching the hallway

**Fig 8:**
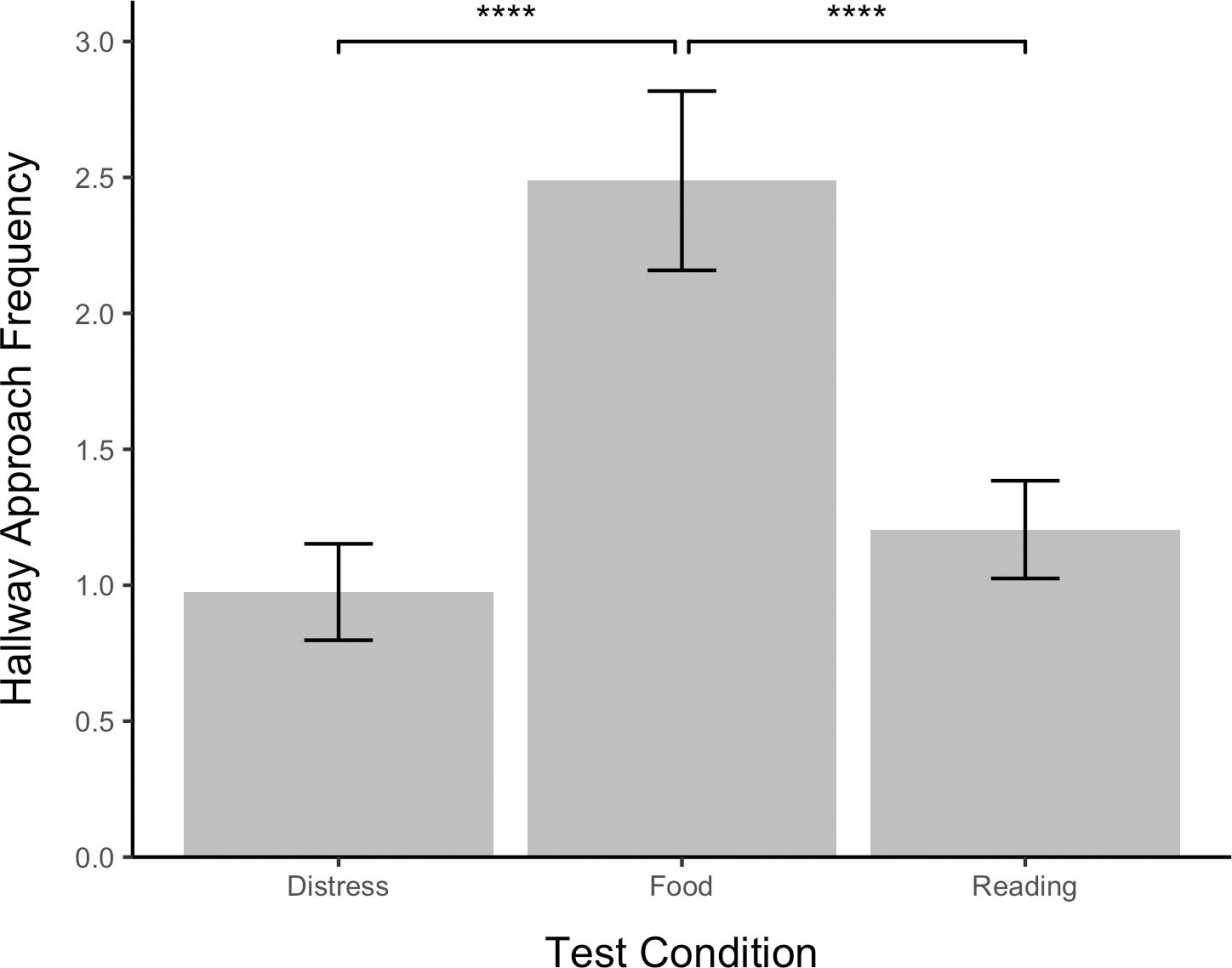
Hallway approach frequency by test condition. Error bars show standard errors.

**Fig 9:**
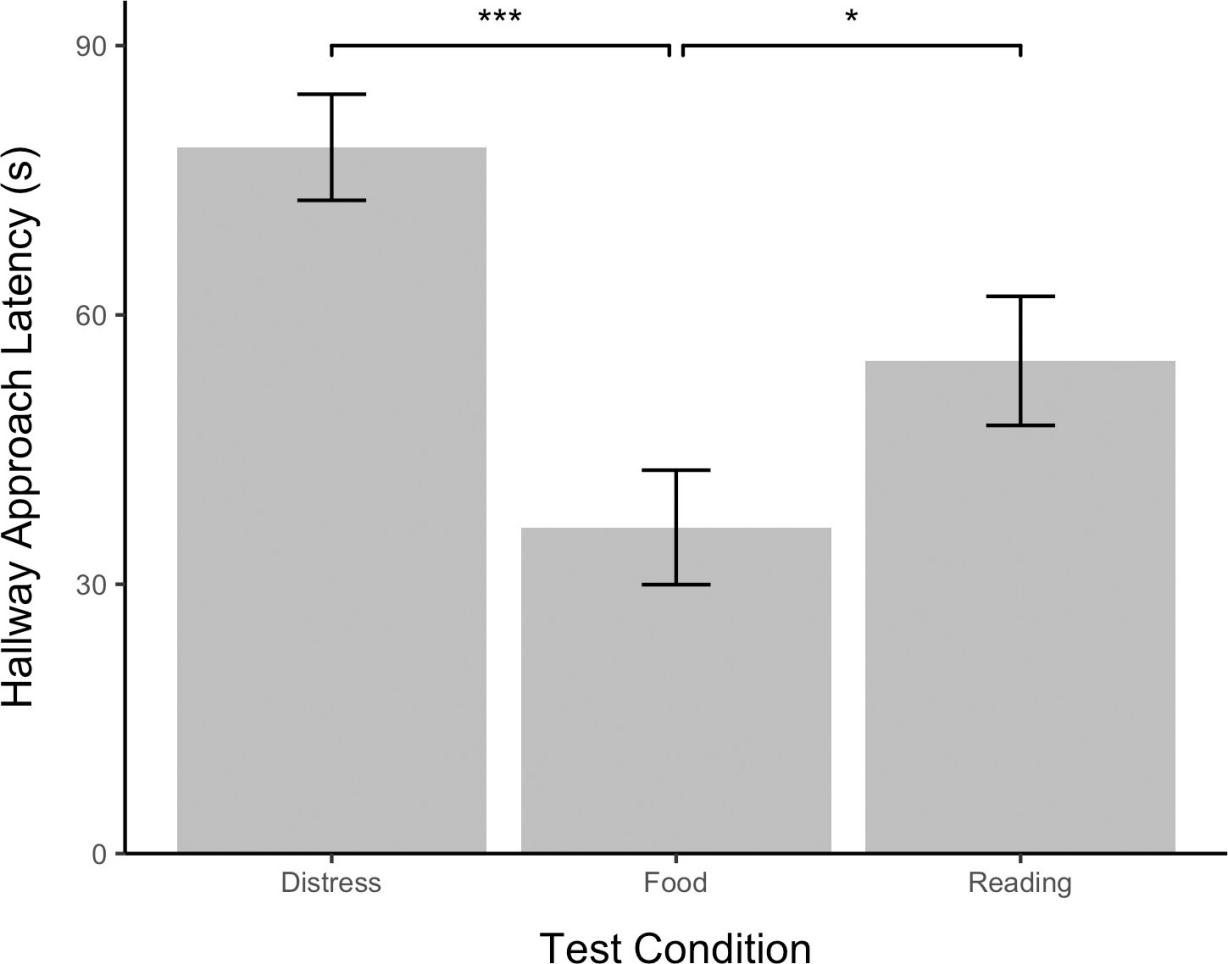
Latency to approach hallway by test condition. Error bars show standard errors.

To assess whether dogs that failed to open the apparatus sought out the experimenter for assistance, the frequency with which the dog approached the hallway and the latency of the dog to first approach the hallway were analyzed using linear regressions. Separate LMMs were constructed for hallway frequency (Fig 8) and latency (Fig 9) in which test number and condition were treated as a fixed-effect predictors and study subjects was treated as a random intercept. Pairwise comparisons among test conditions were then conducted using Tukey post-hoc tests.

Test condition significantly predicted hallway approach frequency, 𝛸^2^(2, *N* = 48) = 39.95, *p* < .0001, and latency, 𝛸^2^(2, *N* = 48) = 27.12, *p* < .0001. Dogs took significantly longer to approach the hallway in the distress test ($\bar{x}$ = 78.67, *SE* = 5.91) than in the reading test ($\bar{x}$ = 78.67, *SE* = 5.91), *z* = 2.80, *p* < .05, but approach frequency in the distress ($\bar{x}$ = .98, *SE* = .18) and reading ($\bar{x}$ = 1.20, *SE* = .18) tests did not significantly differ (*z* = .75, *p* > .05). Hallway approach frequency was greater in the food test ($\bar{x}$ = 2.49, *SE* = .33) than in either the distress test, *z* = 5.80, *p* < .0001, or the reading test, *z* = 5.14, *p* < .0001. Likewise, hallway approach latency was shorter in the food test ($\bar{x}$ = 36.33, *SE* = 6.37) than in either the distress test, *z* = -5.20, *p* < .001, or the reading test, *z* = -2.51, *p* < .05. Test number did not predict hallway approach frequency, 𝛸^2^(2, *N* = 48) = 2.38, p > .05, or latency, 𝛸^2^(2, *N* = 48) = 2.34, *p* > .05. The effect of study subject was significant, 𝛸^2^(2, *N* = 48) = 6.59, *p* < .05.

### Accounting for the staged nature of the rescue scenario

To assess whether the convincingness of the owner’s distress vocalizations affected the performance of dogs in the primary distress test, a GLM and an LMM were used to test the regressions of vocalization score on opening frequency and opening latency, respectively. Vocalization score did not significantly predict opening frequency, 𝛸2(1, N = 60) = .37, p > .05, or latency, 𝛸2(1, N = 60) = .29, p > .05.

### Covariates

To assess whether study subject covariates were adequately controlled through random assignment of test order (DFR, DRF, FDR, FRD, RDF and RFD), separate linear models were used to test the regression of test order on the age, height and weight of the dog. Likewise, a GLM was used to test the regression of test order on the sex of the dog. Test order did not predict sex, 𝛸^2^(5, *N* = 60) = 6.71, *p* > .05, age, *F*(5, *N* = 60) = 2.19, *p* > .05, height *F*(5, *N* = 60) = .25, *p* > .05, or weight, *F*(5, *N* = 60) = .1, *p* > .05.

## Discussion

In the present study, approximately one third of dogs rescued their trapped and seemingly distressed owners, both in the first test (6/20) and across all test orders (20/60). Whether the dog rescued primarily depended on the ability of the dog to complete the task (to open the restrainer apparatus) rather than the dog’s motivation. Among dogs that demonstrated the ability to complete the task in the food test, the propensity for rescue was high (84%). For most dogs, releasing the owner could be attributed to the dog’s separation from the owner in the restrainer apparatus. However, these factors alone could not account for all instances of rescue behavior. Analyses of stress behaviors indicated that owners transmitted their distress to their dogs. This evidence of emotional contagion supports the hypothesis that prosocial motivations contribute to rescue behavior in pet dogs.

### Ability and experience

Cognitive ability and previous experience heavily constrained the performance of dogs in this study. Indeed, dogs that demonstrated the ability to open the apparatus in the food test were eight times more likely to release the owner in either the distress or reading tests. Likewise, when the owner reported that the dog had previously opened objects such as garbage cans, cabinets, dog gates, or containers, the dog was four times more likely to open the apparatus. In addition, the significant effect of test number on opening latency suggested that just one or two exposures to the paradigm altered the tendencies of dogs to open the apparatus.

Rescuing an owner may be a highly rewarding action for dogs. Feuerbacher and Wynne [43] demonstrated that food is a salient reward for dogs, even more so than vocal praise or petting. In the present study, all dogs retrieved treats immediately in both pre-test demonstrations further indicating that these treats served as salient rewards. Thus, the finding that dogs were as likely to release their distressed owner (n = 20) as to retrieve treats from inside the box (n = 19) indicates that dogs were highly motivated to open the apparatus in the distress test.

### Individual variation

Although accounting for performance in the food test helped to control for task ability, our findings indicate that repeated measures were still necessary to control for individual variation among study subjects. Indeed, the random effect of study subject was by far the strongest predictor of opening frequency and latency, stress and activity, and assistance solicitation. Moreover, an average of eighteen dogs opened the apparatus in each of the three successive tests, yet only twenty-four dogs ever opened. Had each dog been tested in only one condition as in Sanford [36] and Carballo [37], the findings of the present study would primarily reflect the test condition assignments of these twenty-four task-capable dogs rather than any differences in the test conditions.

Given that Sanford [36] and Carballo [37] used between-subjects designs and did not test for ability, it is difficult to interpret the findings of these studies. Moreover, these studies did not provide independent evidence that dogs that failed to rescue the owner were cognitively capable of opening the door, even when highly motivated. In addition, only half of the dogs tested in these studies ever opened and the sample sizes of control groups were small. Thus, the assumption that an equal number of task-capable dogs were assigned to the experimental and control conditions may not have been valid. Study designs that do not sufficiently control for individual variation in understanding the contingencies of a prosocial task may lead to misidentification of prosocial behavior [9, 30]. To address this possibility, we suggest that future studies should adopt within-subjects designs and should directly assess the ability of each dog to complete the task.

Additional studies are also needed to clarify the specific experiences and forms of training that influence a dog’s propensity for prosocial rescue behavior. In previous studies, neither nationally-certified therapy dogs [36] nor military search-and-rescue dogs [37] were more likely to release their distressed owners than were pet dogs. However, Carballo et al. [37] found that search-and-rescue dogs released their owners significantly faster than did pet dogs. Although the present study did not compare service and non-service dogs, our findings also suggest that preexisting behavioral tendencies and experiences may constrain performance in the trapped-other paradigm whether or not these experiences were the result of explicit training.

### Motivations for opening

Although task-capable dogs readily released their distressed owner, much of this behavior could be attributed to social contact-seeking, stimulus enhancement, and social facilitation. Indeed, most dogs that released their owners did so regardless of whether the owner conveyed distress. In addition, the owner’s distress did not predict the latency of the dog to release the owner, even when excluding dogs that failed to open for food. It is well established that dogs have evolved hypersocial tendencies which allow for the development of strong bonds with humans [16]. Thus, a strong drive to maintain social contact with humans may preclude the need for, or overshadow, the presence of emotional contagion in prosocial behavior tests for pet dogs.

The role of prosociality in driving rescue behavior in dogs may have been overestimated in Carballo et al. [37] as a result of uncontrolled variables. Relative to emotional contagion, stimulus enhancement and obedience may provide more parsimonious explanations for the observed increase in opening frequency and decrease in opening latency in the experimental group. In the experimental but not the control condition, owners were instructed to scream and were allowed to move and hit the apparatus. These additional stimuli may have increased the likelihood of opening without requiring that the dog perceive the distress of the owner. In the unscripted experimental condition, owners may have also used gestures or words to communicate with, or capture the attention of the dog (e.g., pointing to one’s feet, pretending to hold out a treat or saying “food”, “ball” or “good dog”). Furthermore, pointing to, or hitting near the rock, rope, or door may have aided the dog in releasing the owner by directing the dog’s attention towards the opening mechanisms. These variables may also explain why dogs released the distressed owner more often in Carballo et al. [37] than in the present study.

Given that the primary analyses of Sanford et al. [36] did not rule out alternative explanations for opening, the role of prosociality may have been overemphasized in this study. Specifically, the owner’s distress did not predict opening frequency or opening latency, and in fact, slightly more dogs released the calm owner (n = 11) than the distressed owner (n = 9). Evidence that the owner’s affectual state influenced opening behavior was limited to a small difference in an ad-hoc additional t-test: Considering only the 17 dogs that opened, dogs in the experimental group opened faster than did dogs in the control group. More importantly, it is not clear whether dogs that failed to open (dogs that were omitted from this analysis) lacked the motivation or the ability to open the door. In contrast, in the present study dogs were excluded from the latency analysis based on their performance in a separate assessment for task ability. Ultimately, social contact-seeking, social facilitation, and stimulus enhancement appear to be strong drivers of rescue behavior in the trapped-other paradigm.

### Evidence of prosociality

Although releasing the owner could be attributed primarily to other motivations, multiple lines of evidence indicated that prosocial tendencies represented a significant component of the dog’s motivation to rescue the owner. Indeed, dogs opened more often when the owner called for help than when the owner calmly read aloud. In addition, opening latencies decreased with test number in the distress test but not the reading test suggesting that previous exposures to the paradigm only improved performance when the dog was motivated by the owner’s distress. Furthermore, dogs displayed more stress behaviors in the distress test than in the reading test indicating that owners transmitted their distressed states to their dogs. Therefore, emotional contagion may explain the increase in opening frequency and decrease in opening latencies across tests in the distress test.

The finding that stress scores decreased with test number in the reading test but not in the distress test indicates that dogs experienced different forms of stress in these scenarios. We hypothesize that stress behaviors displayed in the reading test were caused by the dog’s physical separation from the owner while stress behaviors displayed in the distress test were also a product of emotional contagion between the owner and dog. Thus, dogs acclimated to being separated from the owner but the transmission of the owner’s distressed affectual state continued to produce rapid opening in the second and third tests.

Although it is possible that dogs were empathetically motivated to rescue their owner, our findings do not confirm that this behavior was intentionally altruistic. Moreover, emotional contagion during the distress test may have increased the dog’s desire for, or the reinforcing strength of, social contact with the owner. Hollis and Nowbahari [44] argue that, to qualify as rescue behavior, an action must not inherently reward or benefit the rescuer. Therefore, future studies should aim to reduce the potential for immediate egoistic reinforcement. An effective approach may require redesigning the apparatus such that successful rescue of the owner does not result in social contact between the dog and owner (e.g., separated condition in [45]). In such a paradigm, dogs may still rescue the owner to reduce their own stress rather than to reduce the distress of the owner. Moreover, it is not clear that conscious, intentional helping or any form of prosocial behavior can be altruistic in a psychological sense given that such behavior must be reinforcing to the actor in order to be maintained [46, 47] (but see [48]). Notwithstanding these considerations, our findings indicate that emotional contagion of a distressed state increased the likelihood that the dog rescued the owner—a behavior that imposed a cost to the dog, and that instrumentally benefited the owner.

Evidence for possible emotional contagion was unreliable in prior studies. Carballo et al. [37] found that heart rate decreased across repeated trials in control conditions but increased across repeated trials in the experimental condition. However, it is unclear whether dogs in the experimental group became more stressed in response to their owner’s distress or whether they were just more active as a result of obedience and stimulus enhancement. The other four indicators of stress measured in this study indicated that dogs in the experimental and control groups were equally stressed.

Sanford et al. [36] reported that in the experimental group, dogs that released their owner displayed less stress (heart rate variability and stress behaviors) during the test relative to baseline, while dogs that failed to release the owner showed more stress during the test relative to baseline. This interaction between time period (baseline vs. test) and opening was significant in the experimental group but not in the control group. Based on this trend, the authors hypothesized that dogs in the experimental group suppressed their distress responses in order to rescue their owners. However, this difference between conditions (i.e., the interaction between time period, opening *and condition*) was not significant. Moreover, neither test condition nor any interaction involving test condition significantly predicted stress. Thus, reduced stress in the successful openers of the experimental group could not be attributed to the owner’s distressed state. In other words, the assertion that successful dogs suppressed their stress responses relied upon the unsupported premise that these dogs experienced stress responses in the first place.

Future studies should use caution when comparing stress between dogs that did and did not open. Indeed, Sanford’s [36] stress results may reflect the fact that the test durations of openers in the experimental group were significantly shorter than those of openers in the control group. Thus, openers in the experimental condition may not have been tested long enough to display low frequency stress behaviors. Furthermore, the hypothesis that owners transmit their distress to their dogs may not be falsifiable in analyses that treat opening as a predictor of the dog’s stress. Indeed, the opposite outcome of Sanford’s analysis—that successful openers were *more* stressed than non-openers in the experimental condition but not in the control condition—would provide more intuitive support for a positive association between the owner’s distress, the dog’s stress, and prosocial rescue behavior. By assessing the effects of only test number and condition on the dog’s level of stress, the present study avoided this ambiguity as well as potential complications resulting from unequal test durations among openers and non-openers in different test conditions.

### Rescue paradigm consideration

Opening the restrainer door in the present study may have presented dogs with a more challenging task than in previous studies. In Sanford et al. [36], success required that the dog push through a door while walking directly towards the owner. In the present study, the same behavior was not sufficient to open the restrainer door, which could only be moved away from the owner and apparatus. In Caraballo et al. [37], dogs could open the door by pulling a rope, moving a stone doorstop, or inserting their nose or paw into a gap between the door and the lateral wall of the apparatus. Thus, the use of an outward-opening door without multiple opening mechanisms may explain the lower frequency of opening in the present study.

Modifications to the trapped-other paradigm may explain why opening frequencies significantly differed between conditions in the present study and in Carballo et al. [37], but not in Sanford et al. [36]. In line with the original paradigm designed to test rats, in the present study and in Carballo’s study the dog and owner were tested in the same room and were only separated by a thin sheet of material. In addition, both of these studies used repeated measures which allowed successful dogs to learn that opening released the owner. In Sanford et al. each dog was tested only once, in a paradigm that did not include a restrainer apparatus. Moreover, the owner was not confined to a small box, opening the door did not release the owner, and the dog did not have the opportunity to learn the consequence of opening. In addition, this layout separated the owner and dog into different rooms which required that the dog open the door in order to gain close proximity to the owner. This greater spatial separation between the dog and owner in Sanford et al. may have increased the dog’s motivation to open the door, regardless of the owner’s affectual state. Indeed, rescue behavior in the trapped-other paradigm may depend upon the proximity of rescuers to the restrainer apparatus [35, 9]. In support of this hypothesis, opening frequencies in the control condition of Sanford’s study (53%) were substantially higher than in Carballo’s control condition (35%), or the reading test of the present study (27%). Ultimately, these paradigm differences may explain why Sanford et al. [36] did not find a significant effect of condition on opening frequency.

### Staged nature of the distress scenario

Analyses of the owners’ distress vocalizations suggests that the staged nature of the rescue scenario is not a limitation of the current study. Given that the sincerity ratings of the owners did not predict whether the dog rescued, variation in the acting abilities of the owners did not introduce a confound. This finding aligns with previous indications that dogs do not perceive the insincerity of contrived affective states and will respond to feigned crying, anger, and happiness (for review, see [13, 49]). Moreover, in three experiments explicitly designed to test whether dogs detect human deception, Petter et al. [50] found no indications that dogs understand human intentionality. Nonetheless, studies on the prevalence of spontaneous rescue behavior among dogs in genuine emergency situations may provide further insight into the importance of distress sincerity in tests for prosocial rescue behavior.

### Seeking help

The present study did not find any indications that dogs attempted to solicit assistance for their owners from the experimenter standing in the hallway adjacent to the testing room. Instead, approaching the hallway may have been negatively correlated with concern for the owner given that dogs took longer to approach the door in the distress test than in the reading or food tests. In support of this hypothesis, Macpherson and Roberts [27] found that dogs did not seek help from a human bystander but were more attentive towards the owner in two staged emergency scenarios than in a control condition. We suggest that future studies on rescue behavior in pet dogs should focus on instrumental helping rather than help-soliciting behavior.

### Comparisons to rats

Relative to rats, the only other species extensively tested in this rescue paradigm, dogs appear to possess a strong propensity for rescue behavior. Approximately 17% of the rats tested by Ben-Ami Bartal et al. [45] rescued within the first hour of their first trial. Moreover, none of the forty-six rats tested by Tomek et al. [51] opened the restrainer door within the first two minutes of their first trial (Per. Comm.). However, as with dogs, task ability may heavily constrain rescue behavior in rats. Indeed, when trained to open the apparatus in advance of testing, all rats released their trapped cage-mate within the first few trials [52]. Therefore, future studies in which dogs are trained to open the apparatus prior to testing are needed to facilitate further interspecific comparisons.

Rescuing an owner may have been more rewarding for dogs than freeing a cage-mate was for rats. Using a simultaneous choice paradigm, Ben-Ami Bartal et al. [45] showed that rats may find the act of rescuing their cage-mates to be nearly as rewarding as retrieving food treats, and may choose to share food after rescuing cage mates. However, Blystad et al. [52] found that rats waited longer to open the restrainer apparatus and did so less frequently for a trapped cage-mate than for food. In contrast, dogs were as likely to rescue their owner as to retrieve treats.

Our ability to manipulate the apparent affectual state of the owner provided more stringent controls for stimulus enhancement and social facilitation than were possible in previous studies on rats. Although Ben-Ami Bartal et al. [45] demonstrated that the presence of another rat, a toy rat within the restrainer apparatus, and the apparatus itself could not account for observed rescue behavior, the second rat could not be placed in the apparatus in these control conditions. Thus, the degree of stimulus enhancement and potential forms of social facilitation present in the testing scenario were not fully controlled.

Likewise, the reading test allowed us to control for social-contact seeking behavior, an obstacle that has not been fully resolved in rat rescue studies. Although Ben-Ami Bartal et al. [45] demonstrated that rats continued to rescue when social contact was prevented, opening in this control condition may have been a residual response that was not extinguished after the original testing scenario [34, 53]. Indeed, in a follow up study Silberberg et al. [35] found that rats took significantly longer to free trapped cage-mates when social contact was prevented. Furthermore, modifications to the apparatus that rendered successful rescue impossible did not extinguish attempts to open the restrainer, indicating that desire for social contact may still motivate rescue behavior when social contact is prevented.

The transmission of the owner’s distressed affectual state to the dog in the rescue scenario mirrors previous evidence of empathetic concern in rats. Specifically, free rats made more alarm calls when their cage-mate was restrained than when the apparatus was empty or contained a toy rat [45]. In addition, anxiolytic treatment impaired rescue behavior in rats indicating that this behavior was motivated by an affective state of anxiety elicited by the distress of the trapped conspecific [33].

### Future directions

The present study demonstrated that dogs rescue their owners without explicit training and further indicated that dogs capable of completing the required rescue task have a much higher propensity to display prosocial rescue behavior. However, further studies are needed to determine the rescue propensity of dogs that have been trained in advance to perform the required behaviors. Moreover, the extent to which explicit training renders future rescue behavior a pre-conditioned response as opposed to a prosocial behavior resulting from empathetic concern remains unclear. Thus, further research may help to better understand the behavior of working dogs trained in search-and-rescue and related fields.

## Supporting information

S1 Dataset Master dataset. Data are described in the second sheet (“variables”).
(XLSX)

S1 Text Hallway coding. Additional details on the procedure for video coding hallway approaches.
(PDF)
