# Supplementary material for: Pet dogs (Canis lupus familiaris) release their trapped and distressed owners: Individual variation and evidence of emotional contagion
Source: PLoS One. 2020 Apr 16;15(4):e0231742. doi: 10.1371/journal.pone.0231742 (PMC7162277; doi:10.1371/journal.pone.0231742)
Supplement: S1 Text — Additional details on the procedure for video coding hallway approaches. (PDF) [file pone.0231742.s002.pdf]

If the dog was near the hallway when the test began but moved to vacate the hallway area within the first second of the test, the event was not scored as a hallway approach. If, however, the dog did not begin to walk out of the hallway area within three seconds, the event was scored as an approach and a latency of 0.001 seconds was assigned to the test. If the dog did not approach the hallway during the test, a latency of 120 seconds was assigned.
